# Supplementary figures and images for: Taxonomic profiling of Nasutitermes takasagoensis microbiota to investigate the role of termites as vectors of bacteria linked to ironwood tree decline in Guam
Source: PLoS One. 2023 Dec 22;18(12):e0296081. doi: 10.1371/journal.pone.0296081 (PMC10745211; doi:10.1371/journal.pone.0296081)

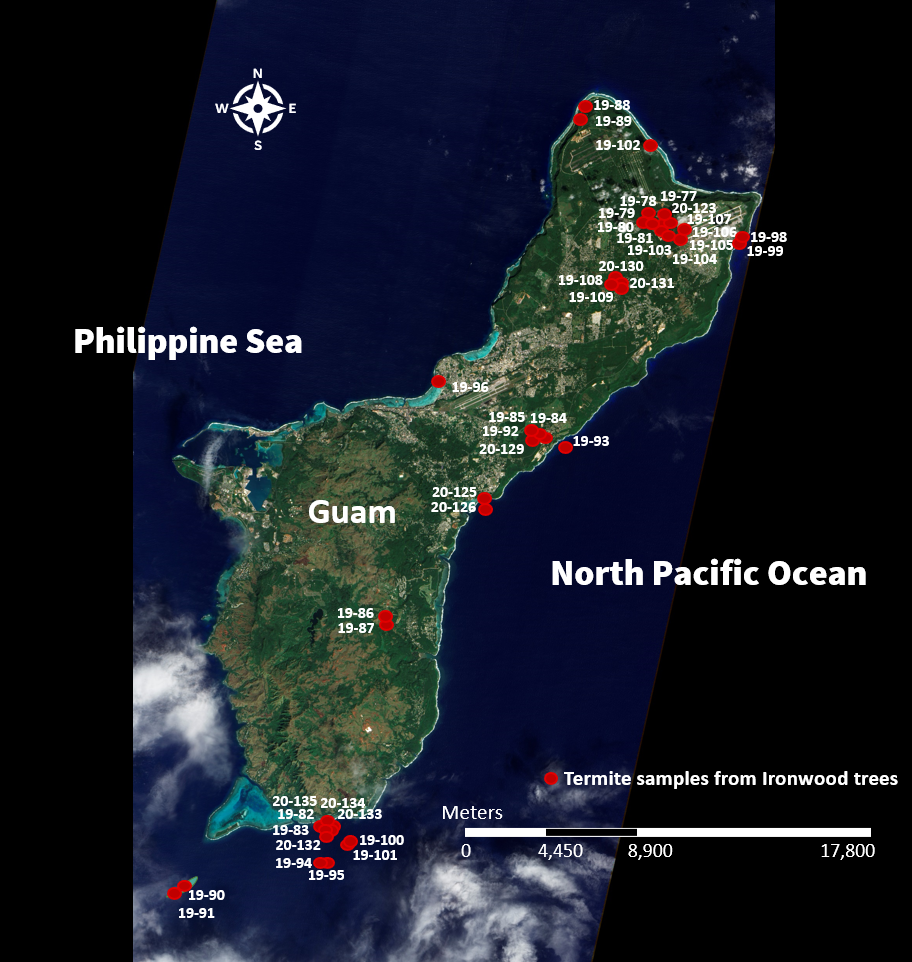

Supplement: S1 Fig — Metadata for each termite sample can be found in S1 Table. (TIF) [file pone.0296081.s001.tif]

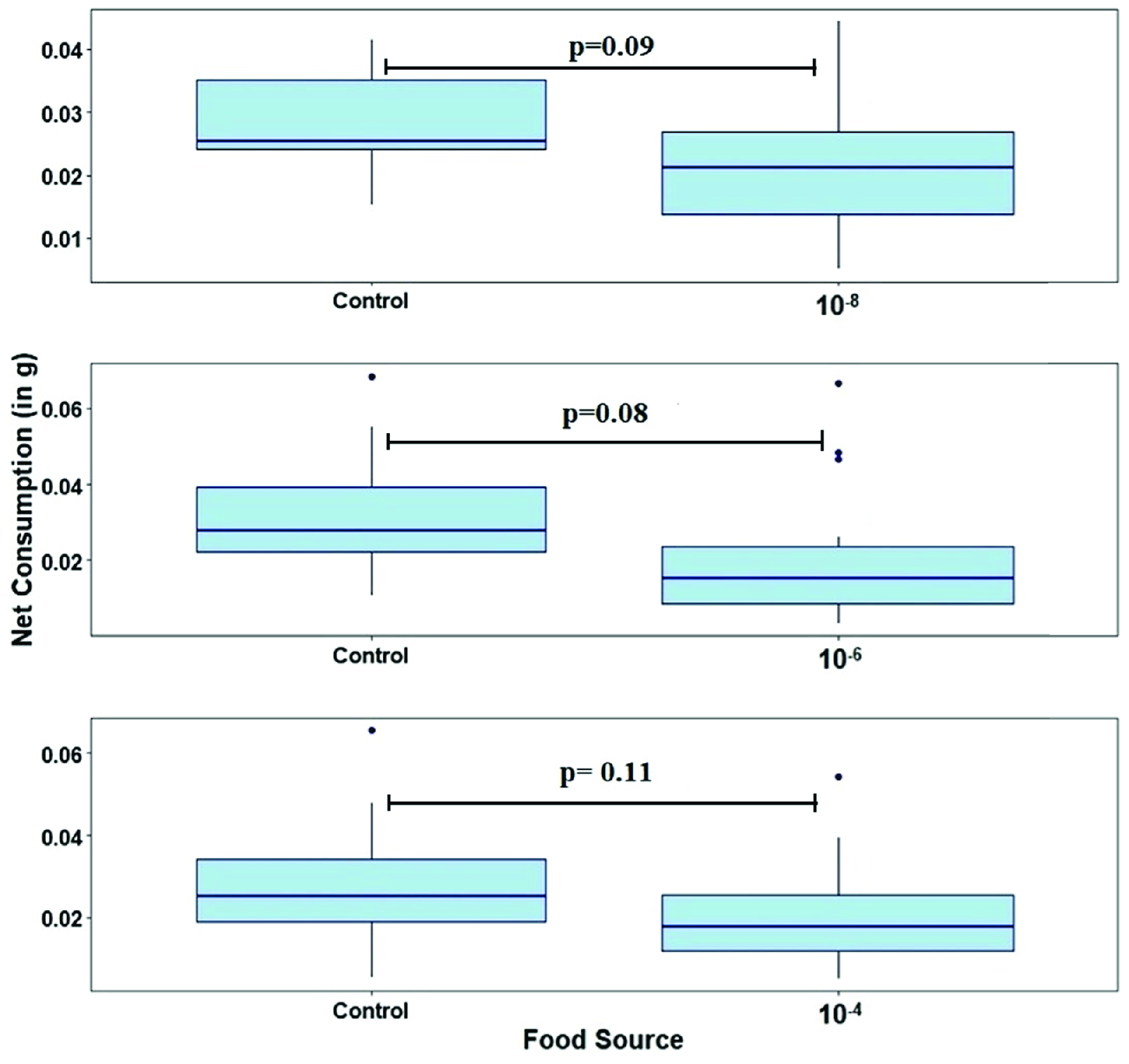

Supplement: S2 Fig — (TIF) [file pone.0296081.s002.tif]

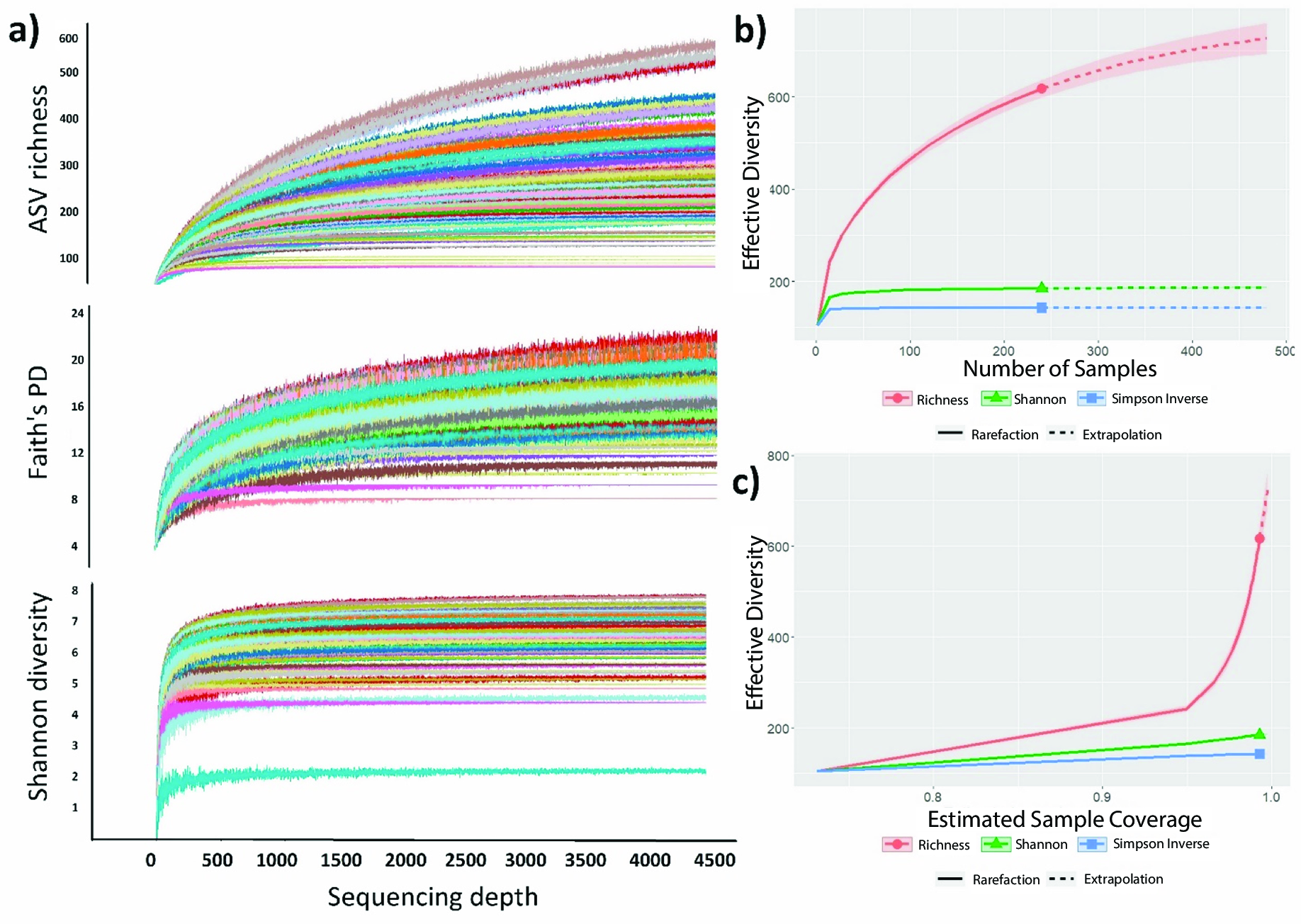

Supplement: S3 Fig — a) Sequence-based rarefaction curves of bacteria diversity showing the number of ASVs (ASV richness), Faith’s phylogenetic distance and Shannon diversity indices for each of the 240 samples of Nasutitermes takasagoensis workers used in no-choice test plotted against sequencing depth. b) Sample-based rarefaction curves across all 240 samples with effective bacterial diversity for different metrics plotted against the number of samples. c) Coverage-based rarefaction curves across all 240 samples with effective diversity plotted against estimated sample coverage. Solid lines indicate intrapolation up to the actual sample size; dashed lines represent extrapolation to twice the sample size. (TIF) [file pone.0296081.s003.tif]

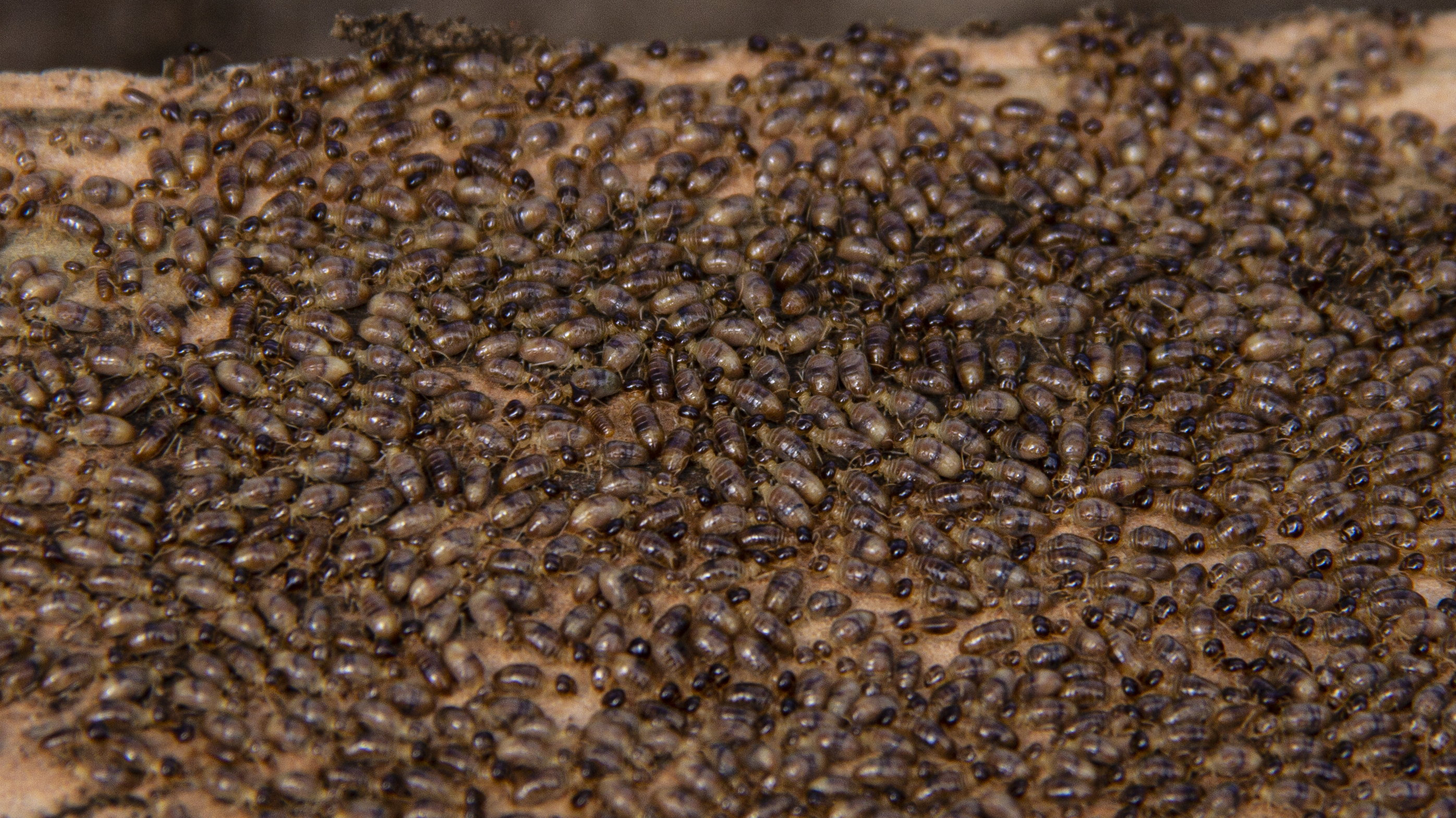

Supplement: S4 Fig — (TIF) [file pone.0296081.s004.tif]
