# Supplementary material for: Taxonomic profiling of Nasutitermes takasagoensis microbiota to investigate the role of termites as vectors of bacteria linked to ironwood tree decline in Guam
Source: PLoS One. 2023 Dec 22;18(12):e0296081. doi: 10.1371/journal.pone.0296081 (PMC10745211; doi:10.1371/journal.pone.0296081)
Supplement: Attachment — Submitted filename: Response to Reviewers.docx [file pone.0296081.s017.docx]

Dear Editor,

Thank you for considering our manuscript entitled “Taxonomic profiling of *Nasutitermes takasagoensis* microbiota to investigate the role of termites as vectors of bacteria linked to ironwood tree decline in Guam” for publication in PLOS ONE. We appreciate the time and effort you and the reviewers took to review our manuscript, and we are pleased to have an opportunity to respond to your concerns.

**Editor's Comments:**

1. Please ensure that your manuscript meets PLOS ONE's style requirements, including those for file naming.

**Response**: We have reviewed the PLOS ONE style requirements and updated our manuscript to adhere to the specified file naming conventions. The manuscript now complies with the provided style templates.

2. In your Methods section, please provide additional information regarding the permits you obtained for the work.

**Response**: No permits were required to conduct the research.

3. PLOS requires an ORCID iD for the corresponding author in Editorial Manager on papers submitted after December 6th, 2016.

**Response**: In compliance with PLOS ONE's requirement, we have included the link to the ORCID iD of the corresponding author within the manuscript.

4. Figure S1 contains an image of a map that may be copyrighted. Replace the figure with a suitable alternative that complies with the Creative Commons Attribution License (CC BY 4.0).

**Response**: We have replaced the previous map with a map sourced from the NASA Earth Observatory, available in the public domain, as suggested. The new map has been appropriately cited in the figure caption.

5. Please keep your tables as part of your main manuscript and remove the individual files.

**Response**: We have integrated the tables into the main manuscript and removed the individual files. Additionally, supplementary tables have been provided as separate spreadsheets in the 'Supplementary Tables FileCH' file.

6. Please review your reference list to ensure that it is complete and correct. Avoid excessive references and prioritize the latest and relevant ones.

**Response**: We have carefully reviewed the reference list to ensure its completeness and correctness and reduced the number of references.

**Reviewer's Comments:**

1. Avoid too many references; prioritize the latest and relevant ones.

**Response**: We appreciate the reviewer's feedback on the number of references used in the manuscript. We have carefully reviewed and revised the manuscript to prioritize the latest and most pertinent references, aligning with the aim of presenting up-to-date and relevant information. Some references were removed.

2. The Introduction and Materials and Methods sections are well written; however, the sentence "We concluded that *N. takasagoensis* workers are not vectors for *Ralstonia* spp. or wetwood bacterial endophytes (*Klebsiella*, *Pantoea*, *Enterobacter*, *Citrobacter*, and *Erwinia*) previously identified in trees symptomatic for IWTD" should be rewritten.

**Response**: We acknowledge the reviewer's positive feedback on the Introduction and Materials and Methods sections. We have carefully reevaluated the mentioned sentence and rephrased it for clarity and precision, in line with the constructive suggestion provided.

We are hopeful that the improvements made to our manuscript align well with the expectations of PLOS ONE. Your valuable insights have significantly contributed to refining our work. We eagerly await your feedback and the possibility of seeing our research published in your esteemed journal.

Best regards,

Garima Setia

Graduate Research Assistant,

Louisiana State University,

Baton Rouge, LA 70803
